# Perspectives on primary eye care

**Published:** 2009-03

**Authors:** GVS Murthy, Usha Raman

**Affiliations:** Senior Lecturer, International Centre for Eye Health, London School of Hygiene and Tropical Medicine, Keppel Street, London WC1E 7HT, UK.; Associate Director and Head, Communications, LV Prasad Eye Institute, Banjara Hills, Hyderabad 500 034, India.

**8^th^ General Assembly of IAPB**

**Course 1:** Primary eye care

**Speakers:** Chad McArthur, Ronnie Graham, Boateng Wiafe, Susan Lewallen, Juan Carlos Silva

**Figure:**
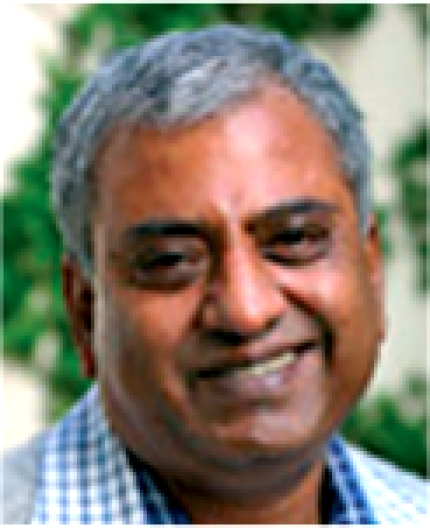


**Figure:**
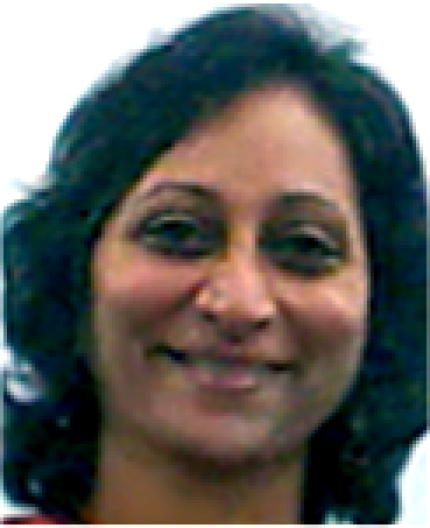


We think of primary eye care (PEC), and any kind of primary health care (PHC), as a ‘frontline’ activity, providing care and identifying disease before it becomes a serious medical issue. However, as this course showed, even a cursory review of systems across the world reveals that there is no common understanding of what primary eye care means and there exists a wide variation both in its content and in the way in which it is delivered.

## The content of primary eye care

Taking a historical perspective, one can trace the initial efforts at establishing PEC to corneal disease, particularly in children, which led to the initiation of vitamin A supplementation programmes, and to trachoma control, which emphasised hygiene and access to water.

In a broad sense, primary eye care is an integral part of comprehensive eye care. It is targeted not only towards preventing blindness and visual impairment, but also towards providing services to redress ocular morbidity.

The box on this page provides a list of components of PEC, but in reality its content can vary greatly, as shown by the speakers' presentations. The discussion focused particularly on the three following aspects:

**Figure:**
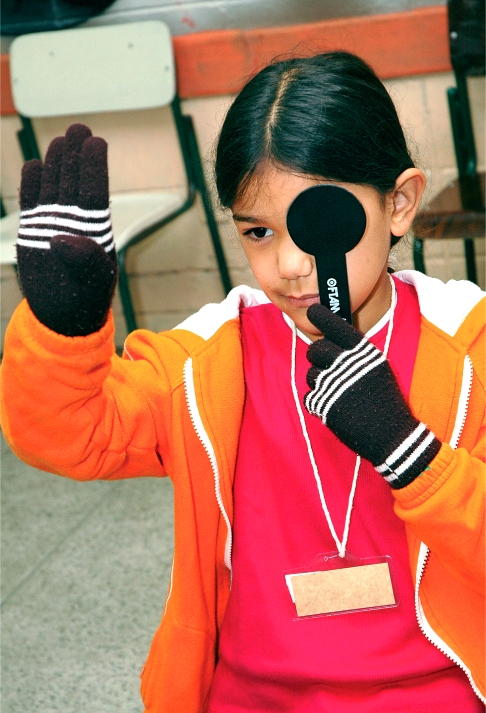
Visual acuity testing in a school. BRAZIL

Fábio Franci

### Timely referral

It was felt that correct identification and timely referral were crucial to the success of PEC. It was stressed that an accurate diagnosis was important for both treatment and referral, as a high proportion of misdiagnosis would lead to a poorer quality of service.

The discussion emphasised the need to provide PEC workers with appropriate diagnostic skills. At the very least, health workers at this level need simple rules to differentiate the serious problems from the ‘trivial’ ones.

### Eye examination

It was acknowledged that the scope of the basic eye examination varies from country to country, depending upon the development of health services. In some countries, it may include ophthalmoscopy, which requires training. PEC in such contexts may necessitate the involvement in service delivery of medical officers or highly skilled eye care workers. However, given the shortage of such trained personnel, this approach limits the spread of PEC and restricts its availability to contexts where such expertise is available.

### Eye health education

It was observed that eye health education was a role for PEC that did not require high-level expertise, in contrast to diagnosis and eye examination. With eye health education, gains can be achieved through awareness and use of basic precautions.

More realistically, therefore, PEC could focus its efforts on eye health education, rather than on diagnosis. PEC workers would construct and deliver messages stressing the importance of diminished vision and the need for modern surgery. They would also allay apprehension or address unfounded beliefs in communities regarding surgery.

## Integration: different approaches to the delivery of PEC

### Integration

Primary eye care cannot be considered as a stand-alone activity but should be integrated into existing primary health care systems (see Table 1).

**Table 1:** Integrating primary eye care into health programmes^a^

Disease	Health programme
Cataract	Healthy ageing
Refractive errors	Healthy schools Healthy ageing
Retinopathy of prematurity	Maternal and child care
Diabetic retinopathy	Non-communicable diseases

As less than 1% of the population is at risk of blinding conditions, it may seem difficult to sustain an entire programme of work around vision problems. However, ocular morbidity is actually much higher, which may be justification enough to allocate more resources to PEC.

Because eye health at the primary care Level is relatively inexpensive and has better return than primary health care in general, it may be easier to set up a primary eye centre than a primary health centre.

**Figure:**
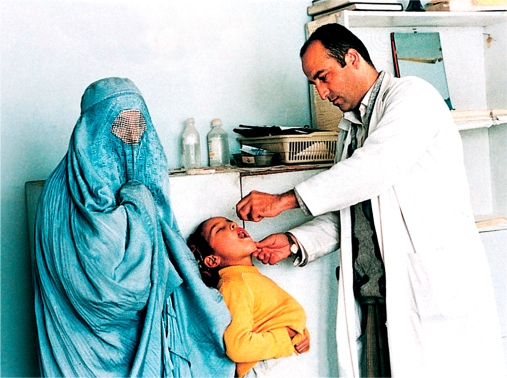
Vitamin A distribution. AFGHANISTAN

M Murtaza Farrahmand

Another approach is to create a hub where multiple health activities can be carried out in coordination.

Different approaches to the delivery of PEC were discussed during the course. Worldwide, experiences with PEC have varied from successful integration with PHC in Thailand, to creating new models of service delivery such as the rural family health system in Pakistan, to vision centres staffed by community workers put through intensive training in screening and identification of basic eye problems.

Drawing on experiences in the field, the presentations made by Ronnie Graham, Boateng Wiafe, and Susan Lewallen focused on PEC in the African context, whilst Juan Carlos Silva discussed the situation in Latin America.

### The experience of PEC in Africa

In Africa, only 30% of people have access to eye care and the spread of available services is uneven across, and even within, countries.

In Kenya, PEC is integrated at all levels of health care through the coordinated efforts of the Department of Prevention and Promotion of Health.

In Gambia, *nyateros* or ‘friends of the eye’ are the first point of contact for PEC. These include community-based rehabilitation (CBR) workers, teachers, and village health workers. These groups help to reduce fear of modern eye care, fight ‘bad’ practices, and generate awareness about eye health.

In Mali and Zimbabwe, the training of PEC workers is done in a cascading manner, while in Zambia training is done at the national level.

The speakers thought that, in Africa, advocacy at the policy-making level was necessary, in order for PEC to be properly integrated into PHC and to be allocated sufficient resources. For PEC to be successful, toolkits need to be developed and some amount of decision making must be allowed at the community level. Efforts should be made to integrate PEC into existing successful programmes, such as the onchocerciasis control programmes.

### Approaches to the delivery of PEC in Latin America

In Latin America, three different approaches have been tried to deliver PEC.

#### Inclusive primary eye care:

In this model, PEC workers were involved in case screening, recognising eye problems, initial treatment, and referral. This approach had a strong component of community participation and was managed by public funding.

However, this system was almost phased out in the 1990s, due to lack of cooperation from PEC workers, ineffective referral mechanisms, and a lack of task-oriented PEC.

#### Selective primary eye care:

In this second approach, specific high-impact interventions were identified and supported under PEC.

The biggest weakness of this approach was that it fostered a vertical system and reduced the opportunity for integration into the existing primary health care systems in countries. This made it unsustainable in the long term.

#### Priority eye conditions:

The third approach focused only on priority eye conditions, identified at national level. Schoolteachers, maternal and child health (MCH) workers, non-communicable disease (NCD) workers and NCD associations were involved. Personnel were trained to handle specific tasks. For instance, schoolteachers were trained to screen for refractive errors.

There is no evidence to indicate which approach is better, but it is clear that different countries may need different approaches.

## Challenges

Challenges to the successful implementation of PEC were discussed during the course. They included:

- determining the type of personnel to be involved
- effectively meeting training needs
- identifying an appropriate mix of skills for effective services in each context
- finding ways to embed PEC in existing health care services without losing focus on eye care.

It was agreed that more scientific evidence was needed on the different modalities of delivering PEC services in developing countries.

Whatever approach is used for PEC, it is imperative that there should be a set of monitoring indicators, which will enable documentation of the strengths and weaknesses of different approaches.

**Primary eye care**

**Definition:** a frontline activity, providing care and identifying disease before it becomes a serious medical issue. Primary eye care can be delivered in many different ways.

**Components of primary eye care:**

- eye health education
- symptom identification
- visual acuity measurement
- basic eye examination
- diagnosis
- timely referral.

**PEC: key points**

1. There is a need to look more critically at the evidence from existing PEC initiatives before we make further investments.
2. PEC can contribute to basic health information systems, as in many areas PEC is the only kind of health care that exists at primary level.
3. It is important to define the skill and knowledge requirements for a PEC worker.
4. PEC should be seen in the context of primary health care (sanitation, nutrition, immunisation, and hygiene).
5. ‘One shoe cannot fit all’: the definition and scope of PEC must vary according to the demands of the context and the design of a country's health system.
6. To be effective, PEC needs a policy framework, relevant structures for service delivery, and adequate financial commitment for implementation.

